# Automatic rehabilitation exercise task assessment of stroke patients based on wearable sensors with a lightweight multichannel 1D-CNN model

**DOI:** 10.1038/s41598-024-68204-1

**Published:** 2024-08-19

**Authors:** Jiping Wang, Chengqi Li, Bochao Zhang, Yunpeng Zhang, Lei Shi, Xiaojun Wang, Linfu Zhou, Daxi Xiong

**Affiliations:** 1 School of Biomedical Engineering (Suzhou), Division of Life Sciences and Medicine, University of Science and Technology of China, Hefei, 230026 China; 2 Suzhou Institute of Biomedical Engineering and Technology, Chinese Academy of Sciences, Suzhou, 215163 China; 3 Neurology Department, Suzhou Xiangcheng People’s Hospital, Suzhou, 215163 China; 4 The First Affiliated Hospital of Nanjing Medical University, Nanjing, 210029 China

**Keywords:** Automatic assessment, Rehabilitation, Wearable sensor, Multichannel, Upper limb action quality, Decision fusion, Biomedical engineering, Rehabilitation

## Abstract

Approximately 75% of stroke survivors have movement dysfunction. Rehabilitation exercises are capable of improving physical coordination. They are mostly conducted in the home environment without guidance from therapists. It is impossible to provide timely feedback on exercises without suitable devices or therapists. Human action quality assessment in the home setting is a challenging topic for current research. In this paper, a low-cost HREA system in which wearable sensors are used to collect upper limb exercise data and a multichannel 1D-CNN framework is used to automatically assess action quality. The proposed 1D-CNN model is first pretrained on the UCI-HAR dataset, and it achieves a performance of 91.96%. Then, five typical actions were selected from the Fugl-Meyer Assessment Scale for the experiment, wearable sensors were used to collect the participants’ exercise data, and experienced therapists were employed to assess participants’ exercise at the same time. Following the above process, a dataset was built based on the Fugl-Meyer scale. Based on the 1D-CNN model, a multichannel 1D-CNN model was built, and the model using the Naive Bayes fusion had the best performance (precision: 97.26%, recall: 97.22%, F1-score: 97.23%) on the dataset. This shows that the HREA system provides accurate and timely assessment, which can provide real-time feedback for stroke survivors’ home rehabilitation.

## Introduction

Stroke is a neurological impairment caused by cerebral vascular accidents or damage to the central nervous system, including cerebral infarction and cerebral hemorrhage^1,2^. It is one of the leading causes of disability and death worldwide. The majority of survivors have varying degrees of motor dysfunction^3^. It seriously affects the activities of stroke patients’ daily lives. Improving limb motor function is a core element of rehabilitation for stroke patients and can greatly improve patient rehabilitation and reduce disability^4^. Exercise-based rehabilitation improves functional ability for people with long-term conditions^5^. Early and intensive rehabilitation, including exercise therapy, can significantly improve motor function and ADLs^6^. Studies have shown that timely and accurate rehabilitation exercises can restore patients’ self-care effectively and that performing individual rehabilitation is helpful for activities of daily living^7^. Stroke assessment is necessary for rehabilitation plans, but most current clinical assessments are based on physician observation and questionnaire scales.

- Fugl-Meyer Motor Assessment (FMA)

The FMA is one of the most popular motor function assessment methods and consists of fifty items for the arm, hand and lower limb, each with three rating scales^8^. FMA has been widely used in clinical practice and research. However, the observation method depends heavily on clinical experience. It is not applicable for patients in a home environment or without the presence of a physician.

- Home-Based Rehabilitation

Throughout the entire rehabilitation period, approximately 92% of the rehabilitation exercises were implemented in the home setting, and 8% were implemented in the hospital setting^9^. Patients need to report on their daily course in the setting and to go to the hospital regularly for progress assessments. It causes an enormous burden on healthcare resources^10^. HBR is a necessary method in which patients perform a series of physical activities at home^11,12^. Patients demonstrated low adherence and motivation for the rehabilitation exercise program, the main influence is the lack of continuous feedback and timely monitoring of exercises in the home setting^13,14^. Therefore, a system that enables home-based rehabilitation training and assessment is urgently needed. The system should meet the following conditions: first, it should be cost-effective, ensure widespread availability without undue financial burden on patients, and be easy to integrate into patients' routines; second, it can provide timely assessments of patients' exercise in the home setting; and third, it should take into account the privacy of the user in the home environment.

- Related Work On Action Quality Assessment

Rehabilitation movement quality assessment is a method that allows a computer to automatically quantify the patient's movement performance and provide interpretable feedback to inform the patient's rehabilitation. It is a specific application of the action quality assessment task^15^ in the medical field. Action quality assessment has received increasing attention in terms of machine learning and computer-aided detection and is widely used in sports and surgery completion. In recent years, action quality assessment has become an important issue in computer-aided applications, such as medical rehabilitation, skill training, and sports activity scoring^16–18^. Research on video-based action quality assessment has focused on the following aspects: detection and preprocessing methods for action^19^, feature extraction methods, and feature methods combined with deep networks^20,21^. Others have started to experiment with deep learning methods for action assessment. Temporal fusion strategies are established by temporal pooling^22^ and temporal relationship learning methods^23^, and significant progress has been made. The disadvantages of the vision-based approach are that using cameras in the home raises privacy issues, the accuracy of motion tracking varies with lighting conditions and clutter, and the ease of use can be limited by space^24^. Thus, the vision-based approach is not applicable in the home environment.

In the home environment, wearable devices can consider both motion signal acquisition and privacy protection. Most of the motion data collected by wearable devices are one-dimensional time series. The processing of one-dimensional data predominantly adopts traditional approaches such as greedy algorithms, Naive Bayes and dynamic time warping^25^. These algorithms are widely used for their simplicity and interpretability, their performance is highly dependent on expertise in feature engineering, which requires a deep understanding of the data's underlying characteristics^26^. In recent years, several studies have attempted to use deep learning methods for one-dimensional signal analysis, such as ECG signals^25,27–29^ and EEG signals^30–32^, and have achieved good results. For other one-dimensional signals, especially motion signals, due to highly individualized data, few studies have focused on this area^4,33^.

Taku Shoji et al*.* described a novel class of compact convolutional neural networks (CNNs) for detecting abnormal patterns and electrodes in EEGs for epilepsy. The designed model is inspired by a CNN developed for brain-computer interfacing called multichannel EEGNet^34^. Yun-Mei et al.^35^ presented a Convolutional Neural Network algorithm to accurately detect EEG abnormalities from multi-channel EEG signals and synthesized several heterogeneous datasets.

Liao et al.^36^ proposed a deep learning framework for rehabilitation training assessment through movement quality output parameters, digitized movement quality assessment equations and a deep neural network (DNN) model with supervised learning and a quality regression approach. Identification was performed by a deep network with a mixture of self-encoder and Gaussian coding and validated by reconstructing the dataset.

Wang et al.^37^ proposed sensor-based activity recognition for deep learning. This study discusses the process of sensor-based recognition, which includes deep models, modalities and applications. Compared with traditional pattern recognition methods, deep learning minimizes the reliance on manual features and achieves improved efficiency.

Hussain^38^ proposed an EMG-based gait monitoring system consisting of a portable EMG device, cloud-based data processing, data analytics, and a health advisor service. This study helps individuals understand stroke-impaired gait changes and aids in poststroke rehabilitation.

Wang et al.^39^ introduced a hybrid multi-feature neural network that combines manually designed features with latent features generated by deep networks. By explicitly considering the motion context and spatio-temporal relations among multiple body parts in the upper limb, the model can detect real-time motions. This study needs to merge data from other body regions for postural calculations to assess the effects of rehabilitation of different body parts. Also, there is a need for an in-depth study of its performance for more fine-grained movements.

Yu et al*.*^40^ proposed a novel remote quantitative FMA framework, in which two accelerometers and seven flex sensors were used to monitor the movement function of the upper limb, wrist and fingers to extend the use of wearable sensor networks for stroke patients' training and assessment in non-clinical settings. This work designed seven training exercises to replace the upper limb-related 33 items in the FMA scale. The work was performed well, but it is difficult for patients with severe hemiparesis to perform these compound movements.

López-Nava et al*.*^41^ proposed a variability analysis of upper limb therapeutic movements using wearable inertial sensors. The data analysis was divided into classification and variability using features and distances calculated previously. This study aims to analyze aspects of the motion of healthy subjects, analysis of groups characterised by limited movement, such as the elderly or patients in rehabilitation is needed.

Some of the above-mentioned studies focused on abnormality detection in EMG or EEG signals as well as human activity recognition. Some studies have concentrated on video-based action quality assessments, which may raise privacy concerns in home settings. Additionally, video-based assessments require users to perform tests in a designated area, while wearable devices are not constrained. Some studies have focused on intelligent Brunnstrom staging methods or attempted to use composite movements for overall FMA assessment. In actual clinical use, users are not willing to complete a full FMA assessment because it takes too much time. Instead, based on prior knowledge of patients’ rehabilitation status, several actions selected from the FMA scale to quickly obtain an assessment of specific actions are needed.

According to the shortcomings listed in the above survey, this study proposes an intelligent home-based rehabilitation assessment framework based on wearable sensing technology and deep learning methods. The main contributions of this study are as follows:

- A wearable rehabilitation exercise system has been developed utilizing motion sensing technology to help patients train in a home environment. The system collects exercise data by placing sensors on specific areas of the body, including the forearm and hind arm. This innovative approach provides a means of collecting physical exercise data for patients undergoing rehabilitation while ensuring their comfort, safety and privacy.
- After adhering to the principles of clinical experimentation and patient voluntarism, a total of 120 participants were recruited to participate in this study. Throughout the experimental period, 1 to 2 additional assessments were conducted based on both patient preferences and physician recommendations. Utilizing the methodology outlined above, a comprehensive rehabilitation assessment dataset was built and analyzed. This approach ensures the collection of high-quality data while also prioritizing patient autonomy and comfort.
- An established method of intelligent feature extraction and analysis is utilized to achieve optimal results. A 1D-CNN model was initially trained on the UCI-HAR dataset. Based on the pretrained model, a multichannel 1D-CNN model is established and tested on our system. By leveraging this approach, we can efficiently evaluate the accuracy and efficacy of the system, ultimately achieving better outcomes.

## Home-based rehabilitation exercise assessment system (HREA)

**Figure 1:**
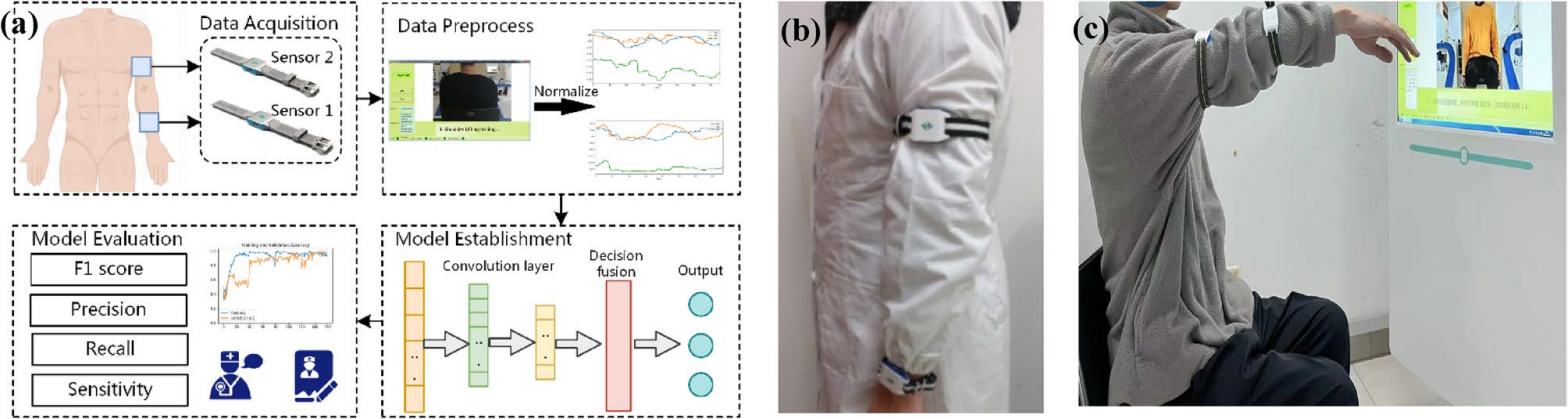
Overall framework of the HREA system: (**a**). overall workflow of the HREA system; (**b**). standard placement of sensors on a physician; (**c**). user trains following standard action guidance.

In this section, the overall architecture of the proposed HREA system shown in Fig. 1 (Fig. 1a shows the overall workflow of the system) is established by the following steps: (a) a rehabilitation exercise and assessment system (consisting of software and wearable sensors) is developed and used to collect rehabilitation data; (b) a basic model is trained on a public dataset and uploaded to the cloud; and (c) a pretrained model stored in the cloud can be used as an initial model for new local tasks. This methodology can significantly reduce training time while concurrently enhancing the model's generalization capabilities and adaptability.

- Overview of System

The whole system consists of three parts: the patient client, the physician client and the web server. Patients wear the wearable sensors first and finish the rehabilitation plans made by physicians. During the exercise period, the sensors record the movement data, transmit it to the computer via the ZigBee protocol and finally upload it from the computer to the web server through the Internet. The sensor is powered by a lithium-ion battery whose capacity is 400 mAh. When the battery is fully charged, the sensor can continuously operate for 10 h. Physicians can view the patients’ training records and Fugl-Meyer assessment via the website and adjust the individualized movement prescriptions according to the assessment. The work primarily focuses on the design of the "patient client" component within a rehabilitation system.

Each wearable sensor consists of an accelerometer, a gyroscope and a magnetometer, which can capture 3D posture data of the human body. The microprocessor unit sensor chip is a mpu9150 with an accelerometer resolution of ± 16 g, a magnetometer resolution of 4800µT and an angular resolution of 2000 degrees per second. These settings enable the measurement of inclination changes less than 0.1 degrees, which is sensitive enough to capture movement features. The sampling frequency of all sensors is set to 30 Hz. We use the Kalman filter to estimate the attitude angle in the sensor and transmit it to the computer, the estimation process is as follows.

Prediction steps:

1 $$\begin{array}{c}{\widehat{X}}_{k|k-1}={F\cdot \widehat{X}}_{k|k-1},\, state\; estimation\end{array}$$

2 $$\begin{array}{c}{P}_{k|k-1}=F\cdot {P}_{k-1|k-1}\cdot {F}^{T}+Q,\, covariance\; matrix\; estimation\end{array}$$

Update step:

3 $$\begin{array}{c}{K}_{k}={P}_{k|k-1}\cdot {H}^{T}\cdot {{(H\cdot {P}_{k|k-1}\cdot H}^{T}+R)}^{-1},\, Kalman\; gain\end{array}$$

4 $$\begin{array}{c}{\widehat{X}}_{k|k}={\widehat{X}}_{k|k-1}+{K}_{k}\cdot ({Z}_{k}-H\cdot {\widehat{X}}_{k|k-1}),\, state\; update\end{array}$$

5 $$\begin{array}{c}{P}_{k|k}=(I-{K}_{k}\cdot H){\cdot P}_{k|k-1},\, covariance\; matrix\; update\end{array}$$

State vector: $X={[\phi , \theta , \psi ,\dot{\phi }, \dot{\theta }, \dot{\psi }]}^{T}$, ϕ, θ, ψ are the roll, pitch and yaw angles, respectively, and $\dot{\upphi }$, $\dot{\uptheta }$, $\dot{\uppsi }$ are the corresponding angular velocities.

Measurement vector: $Z={[{a}_{x},{a}_{y}, {a}_{z},{\omega }_{x},{\omega }_{y},{\omega }_{z},{b}_{x},{b}_{y},{b}_{z}]}^{T}$, ${a}_{x},{a}_{y}, {a}_{z}$ are accelerometer readings, ${\omega }_{x},{\omega }_{y},{\omega }_{z}$ are the gyroscope readings, and ${b}_{x},{b}_{y},{b}_{z}$ are the gyroscope readings.

F is the state transfer matrix, H is the measurement matrix, P is the covariance matrix and I is the initial state.

In this paper, we employ two wearable sensors to conduct the experiment. The hardware cost of the HREA system is less than $50, which can be afforded by patients. The locations of the sensors are shown in Fig. 1b. The sensors were placed in the geometric central area of the upper limbs and elastic bandages were used to keep the sensor firmly attached to the tissue. Sensor 2 (#S2) was placed at a position at least 10 cm away from both the elbow and shoulder, while sensor 1 (#S1) was placed on the forearm within 5 cm of the wrist. We record the attitude angle data of the patient's rehabilitation training and use it as input for the model.

- Assessment Model: Multi-Channel Decision Fusion Model (MCDF)

**Figure 2:**
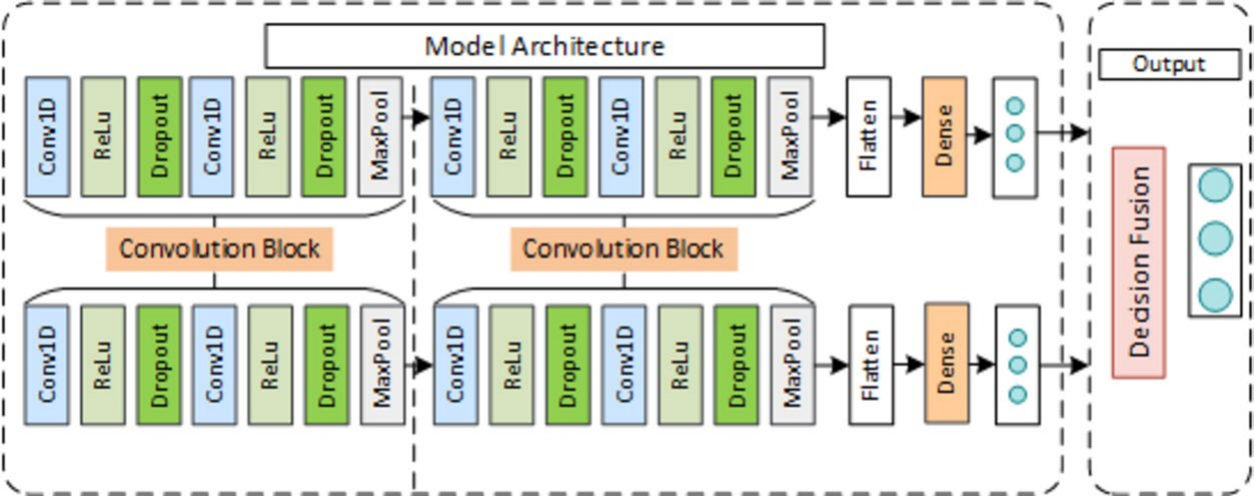
Diagram of the multichannel 1D-CNN architecture for rehabilitation exercise assessment.

A multichannel 1D-CNN model is proposed in this section. As shown in Fig. 2, the assessment model consists of two submodels(1D-CNN) and a decision fusion module. We use the submodel to assess each sensor channel separately and then feed the output predicted values into the decision module as inputs to obtain the final score.

### Preprocessing

To reduce the influence of signal difference, all data obtained from the wearable sensor are normalized to the range of [− 1, 1] using the standard min–max normalization as follows:

6 $$\begin{array}{c}{x}_{i}=\frac{{x}_{i}-{x}_{min}}{{x}_{max}-{x}_{min}}\end{array}$$

where ${x}_{i}$ represents the motion posture value of the i-th sampling point of the action recording $x$. It is worth noting that in this paper, unlike in general normalization, ${x}_{max}$ and ${x}_{min}$ are not the calculated results of each data channel. Ideally, the normalization parameters correspond to the sensor measurement range of each action. In practice, depending on the type of action and the different sensor data channels, these parameters are set to different fixed empirical values.

**Figure 3:**
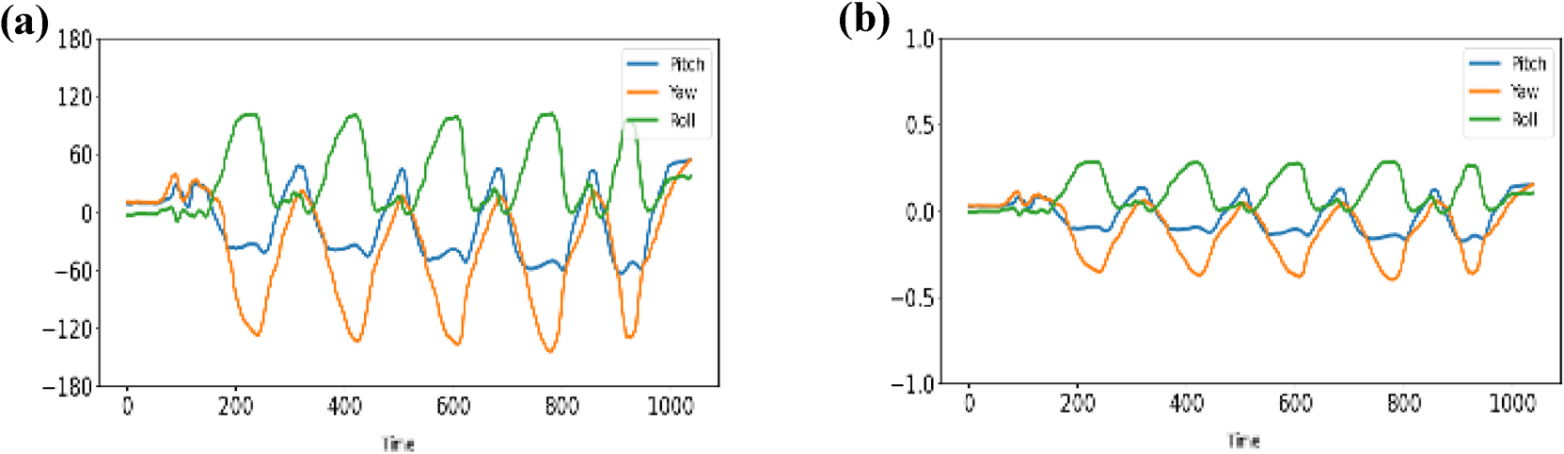
Sensor signals of rehabilitation action: (**a**). raw signals; (**b**). signals after normalization.

Figure 3 shows the signal of the sensor channel corresponding to a complete rehabilitation action captured with the HREA system. Each sensor channel contained 3 channels of attitude angle, as shown in Fig. 7a. The raw signal was normalized before being input into the model using the default sensor range as the normalization parameter (xmax = 180, xmin =  −180). The normalized signal is shown in Fig. 3b. In this way, we do not change the characteristics of the patient's motion signal and, at the same time, we can match our model input range to those in the UCI-HAR dataset, facilitating the training of the rehabilitation assessment model using the parameters of the pre-trained model.

### 1D-CNN module

**Figure 4:**
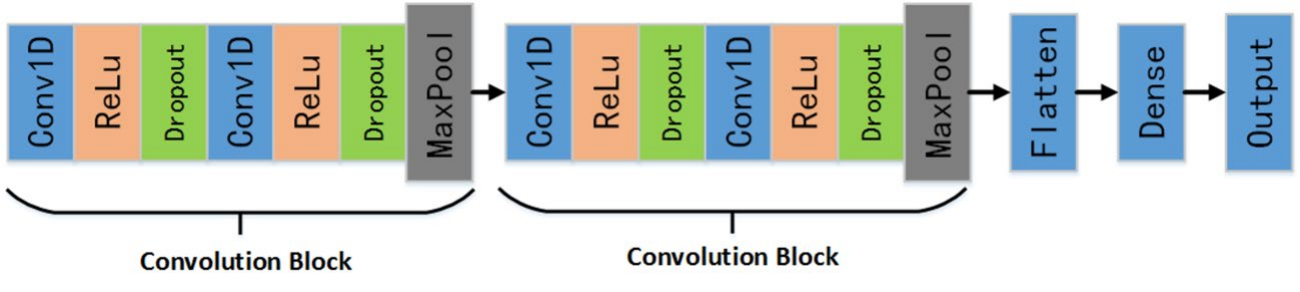
Architecture of each sensor data assessment module.

A 1D-CNN module is established, as shown in Fig. 4. The main roles of the module are: to provide the evaluation results of each sensor channel independently; to act as the backbone of the MCDF model with its category probability vectors as input to the decision layer; to act as the backbone of the MCFF to extract the features of each sensor channel and connect them through the concat layer. The CNN includes two sets of convolution blocks to code local features of action signals, a flattened layer to flatten the feature output of the pooling layer, and a dense layer to build a fully connected layer. Each convolution block contains a convolution layer (Conv1D, kernel size = 10, channel = 3) and a rectified linear unit (ReLU). Then, a dropout layer with a dropout rate of 0.5 is added to the convolution block to avoid overfitting. Finally, a maxpooling 1D layer with a stride of 2 is added after the dropout layer to reduce the size of the features. The network configuration is as follows:

The categorical cross-entropy is used as the loss function to measure the gaps between the predicted values and ground truth. The 1D-CNN module outputs the predicted probabilities.

7 $$\begin{array}{c}Loss=-\sum_{i=1}^{output size}{y}_{i}\cdot log\widehat{{y}_{i}}\end{array}$$

### Decision fusion module

MCDF model uses the decision fusion method to fuse the outputs of two sensors through the 1D-CNN model. Since decision fusion is faster than other fusion techniques, it is suitable for low-cost usage scenarios. Four decision fusion methods are employed to fuse the decision results of multichannel features to obtain an optimal decision strategy suitable for the MCDF model. The decision fusion rules are described as follows.

The max fusion rule is calculated as:

8 $$\begin{array}{c}c=arg\underset{i}{\text{max }}\underset{j}{\text{max}}p\left(\widehat{{c}_{i}}|{x}_{j}\right)\end{array}$$

The sum fusion rule is calculated as follows:

9 $$\begin{array}{c}c=arg\underset{i}{\text{max }}{\sum }_{j}^{n}p\left(\widehat{{c}_{i}}|{x}_{j}\right)\end{array}$$

The Dempster-Shafer (D-S) fusion rule is calculated as:

10 $$\begin{array}{c}c=arg\underset{i}{\text{max }}\frac{1}{K}p\left(\widehat{{c}_{i}}|{x}_{1}\right)\cdot p\left(\widehat{{c}_{i}}|{x}_{2}\right)\end{array}$$

11 $$\begin{array}{c}K=1-{\sum }_{i=0}^{\Omega }p\left(\widehat{{c}_{i}}|{x}_{1}\right)\cdot p\left(\widehat{{c}_{i}}|{x}_{2}\right)\end{array}$$

The Naive Bayes fusion rule is calculated as follows:

12 $$\begin{array}{c}c=arg\underset{i}{\text{max }}p\left(\widehat{{c}_{i}}\right)\prod_{j}^{n}p\left(\widehat{{c}_{i}}|{x}_{j}\right)\end{array}$$

where $c$ denotes the final predicted class, $p(\widehat{{c}_{i}}|{x}_{j})$ is the probability of input ${x}_{j}$ belonging to class $\widehat{{c}_{i}}$ and n is the number of sensor channels. K is the normalization constant, and $\Omega$ is the class variable collection.

- Multi-Channel Feature Fusion Model (MCFF)

**Figure 5:**
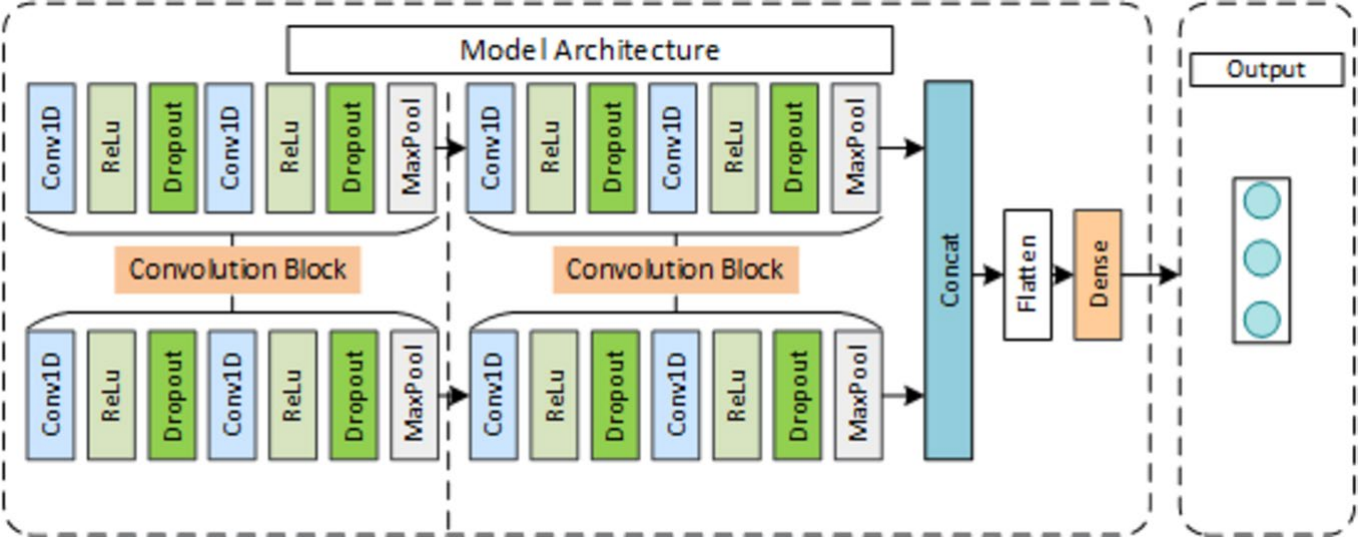
Diagram of the multichannel 1D-CNN feature fusion (abbreviated as F–C) architecture for the HREA system.

In this paper, a multi-channel feature fusion approach model shown in Fig. 5 was proposed as a comparison method to the MCDF model. The feature fusion model consists of two submodules to extract each sensor channel feature, a concatenation function to concatenate the features of each channel, a flattening layer to flatten the features, and a dense layer to build a fully connected layer. The submodel loads initial parameters from the 1D-CNN model pretrained on the UCI-HAR dataset and trains on our dataset. The categorical cross-entropy is used as the loss function. The submodule contains the same two sets of convolution blocks, as shown in Fig. 4.

- Evaluation Metrics

To evaluate the overall performance of the model, the precision, recall and F1 score are used:

The precision is calculated for the model

13 $$\begin{array}{c}Precision\left(P\right)=\frac{TP}{\left(TP+FP\right)}\end{array}$$

where TP is a true positive and FP is a false positive. The recall value is calculated as:

14 $$\begin{array}{c}Recall\left(R\right)=\frac{TP}{\left(TP+FN\right)}\end{array}$$

where FN is the false negative. The F1-Score is calculated as:

15 $$\begin{array}{c}f1 Score\left(f1\right)=\frac{2\times P\times R}{\left(P+R\right)}\end{array}$$

The weighted-avg value is calculated as:

16 $$\begin{array}{c}weighted\; avg(P,R,f1)={\sum }_{n}^{i}{\frac{{N}_{i}}{{N}_{total}}*(P,R,f1)}_{i}\end{array}$$

where ${(P,R,f1)}_{i}$ is the Precision, Recall and $f1 Score$ of the i-th class.${N}_{i}$ is the sample size of i-th class, ${N}_{total}$ is the total sample size. The P, R, F1-score figures below all refer to the weighted average of P, R, F1-score, i is the ith sample classification and n is the total number of sample classifications.

## Experiment

To verify the feasibility of the HREA system, first, a benchmark dataset, the UCI-HAR dataset, was used to train the base model; second, the HREA system was used to collect rehabilitation data to build a clinical rehabilitation exercise and assessment dataset; third, a multichannel 1D-CNN model consisting of two 1D-CNN models built on the UCI-HAR dataset and a decision layer was employed in our system and verified on the built dataset; and, in contrast, a multichannel feature fusion model with two feature extraction sets, a concatenation function, a flattened layer and a dense layer was proposed and verified on the dataset. The model was tested on an ordinary computer with an Intel i5-4590 CPU.

- UCI-HAR Dataset

The UCI HAR dataset contains motion information collected from 30 people of different ages (from 19 to 48 years old) using a Samsung Galaxy SII smartphone attached to their waist. It aims to identify the actions performed by a person through a series of observations of themselves and their surroundings. Several approaches have employed dedicated motion sensors in different body parts, such as the waist, wrist, chest and thighs, to achieve good classification performance^42^. These sensors usually make the average user uncomfortable and do not provide a long-term solution for activity monitoring.

The UCI-HAR dataset uses 72 environmental and human sensors in a sensor-rich environment to record a set of ADLs containing six different types of movements (walking, walking upstairs, walking downstairs, sitting, standing, and lying down), with a total of 2947 data points^43^ shown in Table 1.

**Table 1:** Components of the UCI-HAR dataset.

Activity	Training Samples	Test Samples
Standing	1374	532
Walking	1226	496
Walking Upstairs	1073	471
Walking Downstairs	986	420
Sitting	1286	491
Lying	1407	537

- Dataset Collected With The HREA

**Figure 6:**
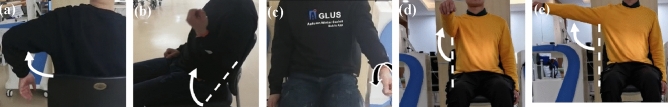
Schematic diagram of the movements selected for rehabilitation: (**a**). shoulder abduction, (**b**). elbow flexion, (**c**). forearm rotation forward, (**d**). shoulder flexion, (**e**). shoulder abduction 90 degrees.

In this study, considering that clinical assessment with the Fulg-Meyer scale is a time-consuming task, we mainly focused on the upper limb motor function of patients, especially the shoulder and elbow, and two wearable sensors were employed in the first stage. The typical actions were selected from the Fulg-Meyer scale for the clinician's assessment. The actions chosen are shown in Fig. 6 and described in Table 2

**Table 2:** Typical (core) set of movements.

Action	Description
shoulder abduction (#A1)	Sit down on a chair, keep the forearm hanging naturally, raise the hemiplegic upper limb along the outer side as high as possible, then hold for 5 s and finally move back to the initial position
elbow flexion(#A2)	Sit down on a chair, keep the upper limb hanging naturally, raise the hemiplegic forearm limb as high as possible, then hold for 5 s and finally move back to the initial position
forearm rotation forward(#A3)	Sit down on a chair, keep the hemiplegic arm straight, and the angle between the arm and trunk is more than 30 degrees rotate the hemiplegic forearm left and right, respectively, and finally move back to the initial position
shoulder flexion (#A4)	Sit down on a chair, keep the hemiplegic arm straight, and raise the arm forward to the horizontal position, then finish the forearm pronation and supination test
shoulder abduction 90 degrees (#A5)	Sit down on a chair, keep the hemiplegic arm straight, and abduce the arm to the horizontal position, then finish the forearm pronation and supination test

The rehabilitation training score labels were evaluated by a clinician and are listed as follows. The clinical scores are 0, 1 and 2, and we add empty labels 3–5 with no feature inputs to keep the same as the pre-training model classification.

17 $$\begin{array}{c}{\text{s}}{\text{c}}{\text{o}}{\text{r}}{\text{e}}\text{=} \, \left\{\begin{array}{l} {0}\text{,} \, {\text{M}}{\text{o}}{\text{v}}{\text{e}}{\text{m}}{\text{e}}{\text{n}}{\text{t}} \, {\text{c}}{\text{a}}{\text{n}}{\text{n}}{\text{o}}{\text{t}} \, {\text{b}}{\text{e}} \, {\text{p}}{\text{e}}{\text{r}}{\text{f}}{\text{o}}{\text{r}}{\text{m}}{\text{e}}{\text{d}} \, \\ {1}\text{,} \, {\text{M}}{\text{o}}{\text{v}}{\text{e}}{\text{m}}{\text{e}}{\text{n}}{\text{t}} \, {\text{i}}{\text{s}} \, {\text{p}}{\text{e}}{\text{r}}{\text{f}}{\text{o}}{\text{r}}{\text{m}}{\text{e}}{\text{d}} \, {\text{p}}{\text{a}}{\text{r}}{\text{t}}{\text{l}}{\text{y}} \, \\  {2}\text{,} \, {\text{M}}{\text{o}}{\text{v}}{\text{e}}{\text{m}}{\text{e}}{\text{n}}{\text{t}} \, {\text{i}}{\text{s}} \, {\text{p}}{\text{e}}{\text{r}}{\text{f}}{\text{o}}{\text{r}}{\text{m}}{\text{e}}{\text{d}} \, {\text{n}}{\text{o}}{\text{r}}{\text{m}}{\text{a}}{\text{l}}{\text{l}}{\text{y}}\\ 3, null \\ 4, null \\ 5, null\end{array}\right.\end{array}$$

The experiment was approved by the relevant hospital ethics committees and was conducted at different hospitals, and patients in various states were involved to ensure data diversity based on compliance to improve algorithmic generalizability. To ensure the balance of different hierarchical data collected in the dataset, the subjects included patients at all stages of rehabilitation, according to Brunnstrom stages II, III, IV, V, and VI of the upper extremity.

Clinical trial registration number: ChiCTR2200061310(date: 20/06/2022), website: https://www.chictr.org.cn/showprojen.aspx?proj=171734.

The 120 included subjects ranged in age from 30 to 80 years, regardless of sex, as shown in Table 3. According to domestic and international clinical studies, stroke patients under 30 years of age have faster recovery and relatively better rehabilitation results than patients over 30 years of age due to their stronger physical functions and willingness to recover, as well as their relatively better ability to receive new rehabilitation technologies.

**Table 3:** General information of participants.

Participants’ information (No. = 120)	Statistical information
Gender: male/female	86/34
Age: mean ± standard deviation	56.5 ± 9.7

Inclusion criteria: (1) Patients with stroke diagnosed by CT or MRI; (2) Age between 30 and 80 years, both sexes; (3) Patients with stable rehabilitation with limb motor dysfunction (hemiplegic motor function evaluation Brunnstrom upper and/or lower extremity grading stage II to VI) caused more than 15 days after stroke onset; (4) Clear cognition and the ability to follow the study protocol; (5) Subjects who understood the purpose of the study, demonstrated adequate compliance with the study protocol and signed an informed consent form.

Exclusion criteria: (1) Significant cognitive or consciousness impairment that prevents completion of the Fugl–Meyer scale; (2) Patients with other significant limb lesions, such as fractures, severe arthritis, amputations, etc.; (3) Formation of joint contractures of the limb; (4) Patients with disabilities prescribed by law, such as blindness, deafness, muteness, mental retardation, psychiatric disorders, and physical disabilities; (5) Patients with combined serious primary diseases, such as cardiovascular, liver, kidney, and hematopoietic system diseases; psychiatric patients(6) Women of childbearing age with positive urine pregnancy test results; (7) Those who participated in other drug/device clinical trials within 90 days before screening; (8) Those who carry active metal devices, such as pacemakers, on their bodies; (9) Other conditions that the investigator considered inappropriate for participation in this trial.

### Ethics approval

This study was approved by the Ethics Committees of Tangdu Hospital (approval number 201912–08) and Xi’an Gaoxin Hospital (2020 ethics review number 001). All methods are carried out following relevant guidelines and regulations. All patients participating in this study have signed the informed consent form. The study is conducted according to the Declaration of Helsinki-ethical principles.

## Results and discussion

**Figure 7:**
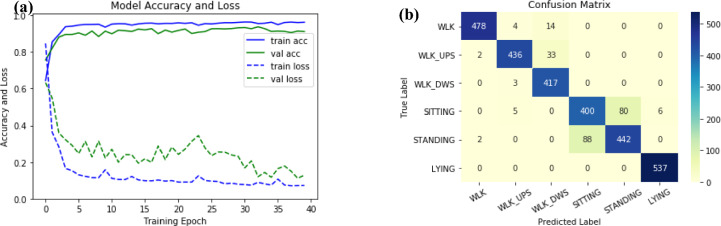
Assessment results of the 1D-CNN on the UCI-HAR dataset: (**a**). accuracy and loss of the network; (**b**). matrix confusion of the network(WLK:Walking, WLK_UPS: Walking Upstairs, WLK_DWS: Walking downstairs).

The 1D-CNN model was trained on the UCI-HAR dataset with a learning rate of 0.01, and the model stabilized after 40 iterations, as shown in Fig. 7a. The classification results are summarized in Fig. 7b and Table 4. The accuracy of the model on the UCI-HAR dataset was 91.96%, which was slightly better than that of the other methods mentioned in Table 4. The 1D-CNN model took 7 ms for a single run on a computer. Based on the pretrained 1D-CNN model, we used this model as the backbone of the proposed multichannel model and verified its effectiveness in our work.

**Table 4:** Performance of the 1D-CNN model with other methods on the UCI-HAR dataset.

Methods	F1-Score (%)
1D-CNN^44^	91.77
2D-CNN^45^	89.53
U-Net^46^	91.33
LSTM-CNN^47^	91.82
Bi-LSTM^48^	84.67
Deep CNN-LSTM with self-attention^49^	91.66
Proposed 1D-CNN Model	91.96

1D-CNN loads parameters from the model pre-trained on the UCI-HAR dataset. We used the 1D-CNN model as a backbone for the MCDF and MCFF models according to the methodology described in the previous section and trained the models on our dataset with a learning rate of 0.001 and a training iteration of 120. A 6:2:2 ratio is used to divide the training, validation and test sets of the built dataset. The assessment results of the 1D-CNN model for each sensor channel were tested separately on the built dataset. The assessment results of the MCFF model based on the feature fusion strategy and the MCDF model based on the decision fusion strategy were compared. In addition, the variability of the results of the MCDF model using four different decisions was analyzed. The results of the 1D CNN model on #S1 and #S2 are used as the baseline, respectively. T-test is used to test the significant difference between models and the significance level is α = 0.05.

Table 5 shows the results of the models over action #A1. The precision, recall, and F1-score evaluation metrics were used to assess the training action assessment results (as described below). We could see from the table that the 1D-CNN model yielded higher evaluation metrics for the #S2 channel (precision: 94.38%, recall: 94.17%, F1-score: 94.17%) than for the #S1 channel (precision: 88.41%, recall: 87.00%, F1-score: 86.92%). As shown in Table 2, action #A1 was used to assess shoulder motor function, which was strongly correlated with the #S2 channel. The #S1 channel was mainly used to collect the movement of the forearm, which had a slightly weaker correlation with action #A1, and ineffective forearm movement also affects the accuracy of action #A1. This was the main reason for the difference in scores between the #S1 channel and the #S2 channel.

**Table 5:** Comparison of the assessment results for #A1 with different fusion methods.

Model	Fusion	Precision (%)	Recall (%)	F1-score (%)	P(< 0.05)	
					Baseline #S1	Baseline #S2
1D-CNN	#S1 with no fusion	88.41	87.00	86.92	–	–
	#S2 with no fusion	94.38	94.17	94.17	–	–
MCFF	Feature concat	89.91	89.95	89.90	0.0370	0.0003
MCDF	MAX	95.98	95.96	95.96	0.0031	0.0013
	SUM	95.98	95.96	95.96	0.0031	0.0013
	**D-S**	**95.98**	**95.96**	**95.96**	**0.0031**	**0.0013**
	Naive Bayes	95.53	95.52	95.52	0.0035	0.0031

Significant values are in bold.

For action #A1, there were statistically significant differences in the performance of the MCDF model compared to the 1D-CNN model for channels #S1 and #S2. The performance of the MCFF model (precision: 89.91%, recall: 89.95%, F1-score: 89.90%) was significantly better than that of the 1D-CNN model for channel #S2 and significantly worse than that of the 1D-CNN model for channel #S1. The MCDF model was optimized for performance (precision: 95.98%, recall: 95.96%, F1-score: 95.96%) using MAX, SUM and D-S evidence-theoretic fusion strategies, with the second-best performance (precision: 95.53%, recall: 95.52%, F1-score: 95.52% ) using the Naive Bayes fusion strategy. We could conclude that the MCDF model with decision fusion strategies fused the information from #S1 and #S2 over action #A1 better than the MCFF model.

Table 6 shows the results of the models over action #A2. The 1D-CNN model performed better for the #S2 channel (precision: 95.08%, recall: 95.07%, F1-score: 95.05%) than for the #S1 channel (precision: 92.54%, recall: 91.03%, F1-score: 91.12%). Action #A2 was used to assess the motor function of the user's elbow joint. The #S2 channel was used to collect the movement signal of the upper arm, while the #S1 sensor was used to collect the movement signal of the forearm. The elbow motion information was calculated from the #S1 channel and #S2 channel. According to the principle of clinical rehabilitation from proximal to distal (from shoulder to forearm), the signal of the #S2 channel was more strongly correlated with that of action #A2 than with that of the #S1 channel, which was consistent with the experimental results.

**Table 6:** Comparison of the assessment results for #A2 with different fusion methods.

Model	Fusion (%)	Precision (%)	Recall (%)	F1-Score (%)	P(< 0.05)	
					Baseline #S1	Baseline #S2
1D-CNN	#S1 with no fusion	92.54	91.03	91.12	–	–
	#S2 with no fusion	95.08	95.07	95.05	–	–
MCFF	feature concat	93.02	92.96	92.90	0.0936	0.0002
MCDF	MAX	95.80	95.52	95.44	0.0090	0.0360
	SUM	95.80	95.52	95.44	0.0090	0.0360
	D-S	96.37	95.96	95.89	0.0057	0.0194
	**Naive Bayes**	**97.32**	**97.31**	**97.31**	**0.0071**	**0.0001**

Significant values are in bold.

Through the statistical test, it could be seen that the performance of MCFF model (precision: 93.02%, recall: 92.96%, F1-Score: 92.90%) was not significantly different from that of the 1D-CNN model on channel #S1, and it was significantly inferior to that of the 1D-CNN model on channel #S2. The performance of the MCDF model was significantly better than those of the 1D-CNN model on channel #S1 and channel #s2. The MCDF model using the Naive Bayes decision strategy performed best (precision: 97.32%, recall: 97.31%, F1-Score: 97.31%).

Table 7 shows the results of the models over action #A3. The 1D-CNN model performed slightly better for the #S2 channel (precision: 92.87%, recall: 92.83%, F1-score: 92.83%,) than for the #S1 channel (precision: 92.54%, recall: 91.03%, F1-score: 91.12%). The model output for each channel was almost consistent, as action #A3 was used to evaluate arm rotation motor function, and both channels could obtain arm rotation motion data.

**Table 7:** Comparison of the assessment results for #A3 with different fusion methods.

Model	Fusion	Precision (%)	Recall (%)	F1-Score (%)	P(< 0.05)	
					Baseline #S1	Baseline #S2
1D-CNN	#S1 with no fusion	92.18	91.93	91.92	–	–
	#S2 with no fusion	92.87	92.83	92.83	–	–
MCFF	Feature Concat	91.39	90.95	90.95	0.0045	0.0058
MCDF	MAX	97.88	97.76	97.76	0.0001	0.0001
	SUM	97.88	97.76	97.76	0.0001	0.0001
	**D-S**	**97.88**	**97.76**	**97.76**	**0.0001**	**0.0001**
	Naive Bayes	96.59	96.41	96.43	0.0001	0.0002

Significant values are in bold.

The performance (precision: 91.39%, recall: 90.95%, F1-score: 90.95%) of the MCFF model was significantly inferior to those of the 1D-CNN model on channels #S2 and #S1. The performance of the MCDF model was significantly better than that of the 1D-CNN model on channel #S1 and channel #s2. The MCDF model using the MAX rule, SUM rule, and D-S evidence theory fusion strategies performed best (precision: 97.88%, recall: 97.76%, F1-score: 97.76%), while the model using the Naive Bayes fusion strategy had the second performance (precision: 96.59%, recall: 96.41%, F1-score: 96.43% ).

Table 8 shows the results of the models over action #A4. The 1D-CNN model performed better for the #S2 channel (precision: 95.74%, recall: 95.52%, F1-score: 95.53%,) than for the #S1 channel (precision: 90.92%, recall: 90.58%, F1-score: 90.60%). Action #A4 was used to assess shoulder flexion ability, and the #S1 and #S channels were used to measure the upper arm and forearm movement signals of action #A4 and to analyze the movement status of the elbow.

**Table 8:** Comparison of The assessment results for #A4 with different fusion methods.

Model	Fusion	Precision (%)	Recall (%)	F1-Score (%)	P(< 0.05)	
					Baseline #S1	Baseline #S2
1D-CNN	#S1 with no fusion	90.92	90.58	90.60	–	–
	#S2 with no fusion	95.74	95.52	95.53	–	–
MCFF	Feature Concat	94.73	94.47	94.38	0.0001	0.0015
MCDF	MAX	96.45	96.41	96.42	0.0003	0.0052
	SUM	96.45	96.41	96.42	0.0003	0.0052
	D-S	96.45	96.41	96.42	0.0003	0.0052
	**Naive Bayes**	**98.66**	**98.65**	**98.66**	**0.0002**	**0.0005**

Significant values are in bold.

The performance (precision: 94.73%, recall: 94.47%, F1-score: 94.38%) of the MCFF model was significantly worse than that of the 1D-CNN model on channel #S2 and significantly better than that of the 1D-CNN model on #S1. The performance of the MCDF model was significantly better than those of the 1D-CNN model on channels #S2 and #S1 and the model using the Naive Bayes fusion strategy performed best (precision: 98.66%, recall: 98.65%, F1-score: 98.66%).

Table 9 shows the results of the models over action #A5. The 1D-CNN model performed slightly better for the #S2 channel (precision: 93.63%, recall: 93.36%, F1-score: 95.53%) than for the #S1 channel (precision: 93.01%, recall: 92.38%, F1-score: 92.49%). Action #A5 was used to assess shoulder abduction ability, and channels #S1 and #S were used to measure shoulder abduction and elbow flexion in action #A5.

**Table 9:** Comparison of the assessment results for #A4 with different fusion methods.

Model	Fusion	Precision (%)	Recall (%)	F1-Score (%)	P(< 0.05)	
					Baseline #S1	Baseline #S2
1D-CNN	#S1 with no fusion	93.01	92.38	92.49	–	–
	#S2 with no fusion	93.63	93.27	93.36	–	–
MCFF	Feature Concat	93.67	93.47	93.38	0.0193	0.2675
MCDF	MAX	95.98	95.96	95.96	0.0031	0.0016
	SUM	98.69	98.65	98.66	0.0009	0.00034
	**D-S**	**98.69**	**98.65**	**98.66**	**0.0009**	**0.00034**
	Naive Bayes	98.21	98.21	98.21	0.0012	0.00051

Significant values are in bold.

The performance (precision: 93.67%, recall: 93.47%, F1-score: 93.38%) of the MCFF model was not significantly different from that of the 1D-CNN model on channel #S2 and significantly better than that of the 1D-CNN model on #S1. The performance of the MCDF model was significantly better than that of the 1D-CNN model on channel #S1 and channel #s2. The MCDF model using the SUM rule, and D-S evidence theory fusion strategies performed best (precision: 98.69%, recall: 98.65%, F1-score: 98.66%), while the model using the Naive Bayes fusion strategy had the second performance (precision: 98.21%, recall: 98.21%, F1-score: 98.21%).

Table 10 shows the performance of the multichannel model on the five action sets in comparison to that of the 1D-CNN model. The performance (precision: 94.34%, recall: 94.17%, F1-score: 94.19%) of 1D-CNN on channel #S2 was better than that of the model on channel #S1 (precision: 91.41%, recall: 90.58%, F1-score: 90.61%). This was because the channel #S2 signal was the proximal signal of the upper limb, which was less disturbed and strongly correlated with the selected actions; the channel #S1 signal was the distal signal of the upper limb, which was more disturbed and weakly correlated with some actions.

**Table 10:** Comparison of the assessment results for all actions with different fusion methods over the 1D-CNN model for the #S1 and #S2 channel.

Model	Fusion	Precision (MEAN) (%)	Recall (MEAN) (%)	F1-Score (MEAN) (%)	P(< 0.05)	
					Baseline #S1	Baseline #S2
1D-CNN	#S1 with no fusion	91.41	90.58	90.61	–	–
	#S2 with no fusion	94.34	94.17	94.19	0.0429	–
MCFF	Feature Concat	92.54	92.36	92.30	0.2214	0.0856
MCDF	MAX	96.42	96.32	96.31	0.0005	0.0107
	SUM	96.96	96.86	96.85	0.0007	0.0092
	D-S	97.07	96.95	96.94	0.0006	0.0060
	**Naive Bayes**	**97.26**	**97.22**	**97.23**	**0.0004**	**0.0041**

Significant values are in bold.

The performance of the MCFF model was not significantly different from 1D-CNN over the selected actions on both channels #S1 and #S2. The performance of the MCDF model using different decision strategies was significantly better than that of the 1D-CNN model on both channels #S1 and #S2 over the selected actions. The MCDF model using the Naive Bayes decision strategy had the best performance (precision: 97.26%, recall: 97.22%, F1-score: 97.23%) on the set of actions. It could be seen that the MCDF model fused the multi-channel information well and the performance improvement was significant.

The time cost of the models was tested on an ordinary computer with an Intel i5-4590 CPU, as shown in Table 11. The time consumption of the 1D-CNN for each sensor channel was 7 ms. The MCFF model consumed 23 ms for a single run. The MCDF model consumed 18 ms for a single run, and there was no significant difference in the time consumption of the MCDF model with respect to the decision fusion strategies used in the work. A processing speed of more than 30 times per second was sufficient to meet the family's use needs. Both of the above methods were successful, but the processing speed of the MCDF model was better than that of the MCFF model.

**Table 11:** Comparison of the time consumption of models.

Model	Fusion	Time–Cost (ms)
1D-CNN on #S1	–	7
1D-CNN on #S2	–	7
MCFF	Feature Concat	23
MCDF	MAX	18
	SUM	18
	D-S	18
	Naive Bayes	18

## Conclusion

In this paper, we built a home rehabilitation exercise assessment system (HREA) based on wearable sensors for the home environment and established a multichannel model using decision fusion to assess rehabilitation action. The system was sufficiently inexpensive and required very little computing resources. The 1D-CNN model was first built and trained on the UCI-HAR dataset, and its F1-score reached 91.96%, which was slightly better than that of the literature methods. Following the guidance of a clinical rehabilitation specialist, five common clinical core upper limb actions selected from the FMA scale were used to construct the dataset to validate the system. Based on the 1D-CNN model, we built a multichannel model using a decision fusion strategy to assess the quality of rehabilitation exercise. We compared the performances of the multichannel model (MCDF) using four different decision fusion strategies, the 1D-CNN model for each sensor channel, and the multichannel model (MCFF) using the feature fusion strategy. The MCDF model significantly outperforms the baselines, while the MCFF model shows no significant improvement over the baselines. The MCDF model using the Naive Bayes decision strategy had the best performance (precision: 97.26%, recall: 97.22%, F1-score: 97.23%) on the set of actions. The MCDF model took 18 ms for a single run on an ordinary computer with an Intel i5-4590 CPU, which ensured that the HREA system would work properly on home computers.

Additional work on the assessment of the Fugl-Meyer scale based on this method for other actions is in progress for further validation of the broad validity of this method. We plan to develop more sensor channels for the acquisition of whole-body movement signals. In addition, by further streamlining the system, we plan to deploy the system at the edge node without a computer, making it easier to use.

## Data Availability

The data are available at https://github.com/rodddy/RehabilitationData.
